# Monocrotaline Toxicity Alters the Function of Hepatocyte Membrane Transporters in Rats

**DOI:** 10.3390/ijms23147928

**Published:** 2022-07-19

**Authors:** Catherine M. Pastor, Valérie Vilgrain

**Affiliations:** 1 Department of Radiology, University Hospital of Geneva, 1205 Geneva, Switzerland; 2 Centre de Recherche sur L’inflammation, Inserm, U1149, CNRS, ERL8252, 75006 Paris, France; valerie.vilgrain@aphp.fr; 3 Department of Radiology, Hôpital Beaujon, Hôpitaux Paris Nord Val de Seine (AP-HP), France Université de Paris, 92110 Clichy, France

**Keywords:** sinusoidal obstruction syndrome, hepatocyte transporters, perfused rat livers, hepatocyte concentrations, clearances

## Abstract

Pyrrolizidine alkaloid monocrotaline (MCT) induces sinusoidal obstruction syndrome (SOS) in rats characterised by a sinusoidal congestive obstruction. Additionally, MCT administration decreases the biliary excretion of gadobenate dimeglumine (BOPTA), a hepatobiliary substrate used in clinical imaging. BOPTA crosses hepatocyte membranes through organic anion transporting polypeptides, multidrug-resistance-associated protein 2, and Mrp3/4 transporters, and a modified function of these transporters is likely to explain the decreased biliary excretion. This study compared BOPTA transport across hepatocytes in livers isolated from normal (Nl) rats and rats with intragastric administration of MCT. BOPTA hepatocyte influx clearance was similar in both groups, while biliary clearance and bile concentrations were much lower in MCT than in Nl livers. BOPTA efflux clearance back to the sinusoids compensated for the low biliary excretion, and hepatocyte concentrations remained similar in both groups. This SOS-associated changes of transporter functions might impact the pharmacokinetics of numerous drugs that use similar transporters to cross hepatocytes.

## 1. Introduction

Understanding hepatocyte concentrations of substrates is a recent translational topic. In the past, great achievements in the understanding of substrate distribution in the liver have been primarily made through the measurement of plasma concentrations. Liver concentrations were not available. Therefore, when conducting pharmacokinetic studies, the classical approach assumed that unbound concentrations approximate unbound plasma concentrations. With the emerging knowledge of hepatocyte membrane transporters, it became clear that, depending on the relative hepatocyte influx and efflux clearances of substrates, unbound hepatocyte concentrations can exceed, equal, or be lower than unbound plasma concentrations. Disconnection between hepatocyte and plasma concentrations of various endogenous or exogenous substrates becomes even more unpredictable when the expression and functions of membrane transporters are altered during liver diseases [1,2]. With advances in imaging, the quantification of liver concentrations is now possible following the injection of hepatobiliary substrates [3]. Using pharmacokinetic modelling, liver imaging can also predict the function of influx and efflux transporters of hepatocytes [4].

Most patients with liver metastases from colorectal cancer receive chemotherapy before liver resection. This presurgical chemotherapy may induce liver injuries such as steatosis, nodular regenerative hyperplasia, steatohepatitis, and sinusoidal obstruction syndrome (SOS) [5]. This chemotherapy-associated liver injury increases the number of complications following hepatectomy. Post-chemotherapy SOS is characterised by the loss of sinusoidal wall integrity and subsequent sinusoidal congestive obstruction. SOS regresses only months after the end of chemotherapy [6]. The syndrome has been associated with more than 20 drugs, including conventional immunosuppressive and chemotherapeutic agents such as oxaliplatin.

Chemotherapy in rodents does not induce SOS, but the syndrome is observed after monocrotaline (MCT) gavage [7]. MCT is a pyrrolizidine alkaloid metabolised into the toxic compound monocrotaline pyrrole by cytochrome P450 enzymes present in sinusoidal endothelial cells, inducing sinusoidal injury. In this model, we previously showed that sinusoidal obstruction persists 18 days after MCT gavage in 25% of rat livers [8]. Following a 70% hepatectomy, liver regeneration was significantly impaired, and this impaired regeneration was associated with hepatocellular injury. The biliary excretion rates of the imaging substrate gadobenate dimeglumine (BOPTA) was significantly decreased.

**Figure 1:**
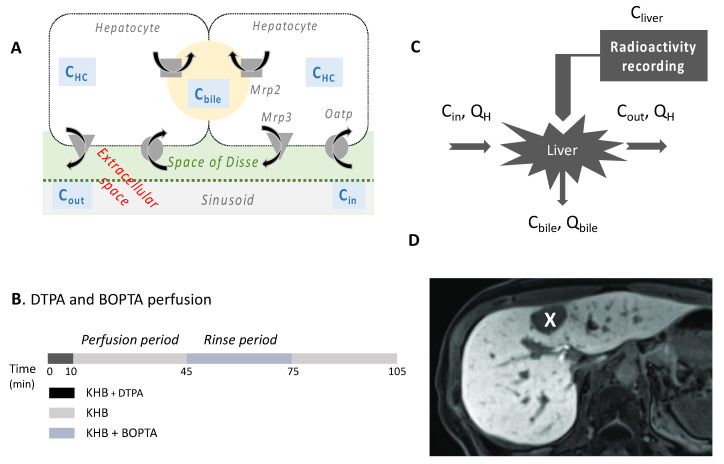
(**A**) BOPTA enters hepatocytes through the rat organic anion-transporting polypeptides (Oatps) and exits from hepatocytes into the bile canaliculi through the multidrug-resistance-associated protein 2 (Mrp2) and back into the sinusoids through Mrp3/4. (**B**) Two imaging substrates, DTPA and BOPTA, are subsequently perfused in isolated rat livers. DTPA distributes within the extracellular space (sinusoids and space of Disse), while BOPTA distributes into the extracellular space, hepatocytes, and bile canaliculi. Both substrates were diluted in a Krebs–Henseleit bicarbonate (KHB) solution. (**C**) Portal vein was perfused with 200 µM BOPTA (C_in_) at a liver flow rate (Q_H_) of 30 mL/min. In the common bile duct, BOPTA concentrations (C_bile_, µM) and bile flow rates (Q_bile_, µL/min per liver) were measured. During the perfusion period, BOPTA concentrations in hepatic veins (C_out_, µM) were concentrations that did not enter hepatocytes plus concentrations that entered hepatocytes before returning to the sinusoids. During the rinse period, C_out_ are concentrations leaving hepatocytes because no BOPTA was perfused. DTPA and BOPTA liver concentrations were measured with a gamma counter placed over a liver lobe. (**D**) BOPTA characterises focal lesions in humans. BOPTA enters normal human hepatocytes (white areas) while BOPTA is not present in the tumour (black area, white cross) because they do not possess uptake transporters. Gadopentetate dimeglumine (DTPA; Magnevist^®^; Bayer imaging) and gadobenate dimeglumine (BOPTA, MultiHance^®^; Bracco Imaging).

BOPTA is a hepatobiliary substrate able to characterise human liver function and focal lesions when injected during magnetic resonance imaging (MRI) (Figure 1D) [9,10]. This characterisation relies on BOPTA transport across hepatocyte membrane proteins. We previously showed in cultured oocytes that BOPTA is a substrate of the rat organic anion-transporting polypeptides (Oatps) (Figure 1A) [11]. BOPTA is not excreted in the bile canaliculi of rats lacking the multidrug-resistance-associated protein 2 (Mrp2) [12]. We also showed that BOPTA is excreted back into sinusoids, but we did not identify the responsible membrane transporters. Mrp3/4 transporters might be involved as published with another imaging substrate, gadoxetate dimeglumine [13]. In the present study, we characterised how the alterations of transporter functions in injured hepatocytes explain the decreased biliary excretion of BOPTA associated with SOS.

## 2. Results

### 2.1. Basic Pharmacokinetic Parameters

**Figure 2:**
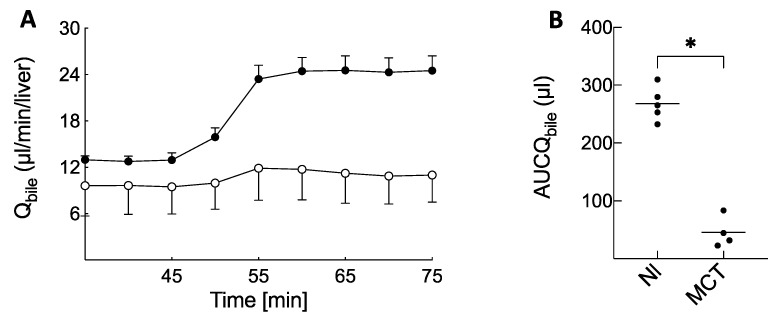
(**A**) Bile flow rates (Q_bile_) during BOPTA perfusion (45 to 75 min). Livers were perfused with Krebs–Henseleit bicarbonate solution (KHB) + 200 µM [^153^Gd]BOPTA. Normal liver (Nl, black circles) and MCT livers (white circles). (**B**) Area under the curve of Q_bile_ (AUCQ_bile_) increase during BOPTA perfusion. * *p* = 0.02.

Before BOPTA perfusion, bile flow rates (Q_bile_) were 13.0 ± 0.9 (Nl livers) and 9.5 ± 3.5 µL/min per liver (MCT rats) (*p* = 0.10, Figure 2A, before BOPTA perfusion). During BOPTA perfusion, BOPTA Q_bile_ increased. This choleretic effect, measured by the area under the curve of Q_bile_ increase, was lower in MCT (46 ± 27 µL) than in Nl livers (268 ± 29 µL, *p* = 0.02, Figure 2B). BOPTA has a low liver extraction ratio, and this ratio was significantly decreased in MCT (4 ± 1%) versus Nl (8 ± 1%) livers (*p* = 0.02).

### 2.2. BOPTA Accumulation in Liver Compartments

**Figure 3:**
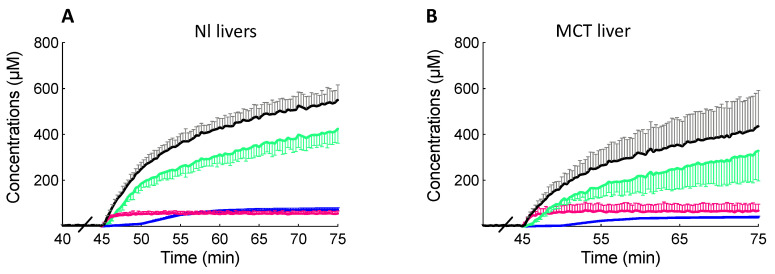
BOPTA compartmental distribution in normal (Nl, **A**) and MCT livers (**B**). Livers were perfused with Krebs–Henseleit bicarbonate solution (KHB) + 200 µM [^153^Gd]BOPTA (45 to 75 min). Liver concentrations (black symbols) were measured by a gamma counter. Concentrations in extracellular compartment (red symbols) were measured during DTPA perfusion. Concentrations that originate from bile canaliculi (blue symbols) and from 78% hepatocytes (green symbols) were calculated.

In the liver, BOPTA accumulates in the extracellular space, hepatocytes, and bile canaliculi (Figure 1A). The extracellular concentrations (C_EC_) over time were not significantly different in MCT and Nl livers (*p* > 0.99, Figure 3 and Table 1). Hepatocyte concentrations (C_HC,78%_) were also similar over time in both groups (*p* > 0.99, Figure 3 and Table 1). In contrast, concentrations measured by the counter in bile canaliculi (C_BC_) over time were significantly lower in MCT than in Nl livers (*p* < 0.0001, Figure 3 and Table 1). Finally, liver concentrations (C_liver_) were also similar over time in both groups (*p* > 0.99, Figure 3 and Table 1).

### 2.3. Accumulation Profile of BOPTA Concentrations in Hepatocytes

**Figure 4:**
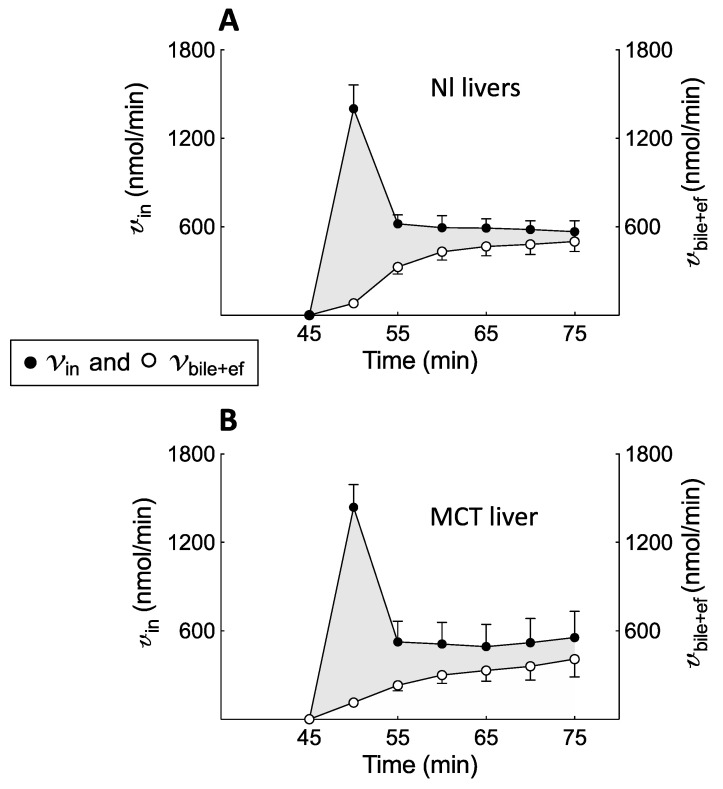
BOPTA hepatocyte influx rates (v_in_, left *Y-axis*, black circles) and bile plus basolateral efflux rates (v_bile+ef_, right *Y-axis*, open circles) during BOPTA accumulation (45 to 75 min) in normal (Nl) (**A**) and MCT (**B**) livers. Livers were perfused with Krebs–Henseleit bicarbonate solution (KHB) + 200 µM [^153^Gd]BOPTA. Difference between v_in_ and v_bile+ef_ (grey area).

The accumulation profile of BOPTA concentrations in hepatocytes relied on the concomitant influx rates (v_in_, nmol/min) and efflux rates (v_bile+ef_, nmol/min). v_in_ over time was not significantly different in MCT and Nl livers (Figure 4 and Table 1). In contrast, bile excretion rates (v_bile_) over time were significantly lower in MCT than in Nl liver (*p* < 0.0001, Table 1). In contrast, efflux rates into sinusoids (v_ef_) were significantly higher in MCT than in Nl livers (*p* < 0.0001, Table 1). In both groups, influx rates were higher than total efflux rates (Figure 4, grey area), and the difference was low at the end of the perfusion period.

BOPTA influx rate into hepatocytes (v_in_) was defined by [C_in_ − (C_out_ − C_ef_)] × Q_H_. C_ef_ is BOPTA concentration leaving the hepatocytes. This influx rate differs from v defined by (C_in_ − C_out_) × Q_H_, which quantifies BOPTA removal rates from livers. v_in_ is higher than v and includes v_ef_ or efflux rate from hepatocytes to sinusoids. The liver removal rate was significantly lower in MCT than in Nl livers over time (Table 1, *p* < 0.0001). C_out_ was only slightly lower than the portal vein concentrations (C_in_), reflecting the low liver extraction ratio of BOPTA. Moreover, C_out_ at the end of BOPTA perfusion was significantly higher in MCT (192 ± 2 µM) than in Nl livers (184 ± 2 µM, *p* = 0.02).

**Figure 5:**
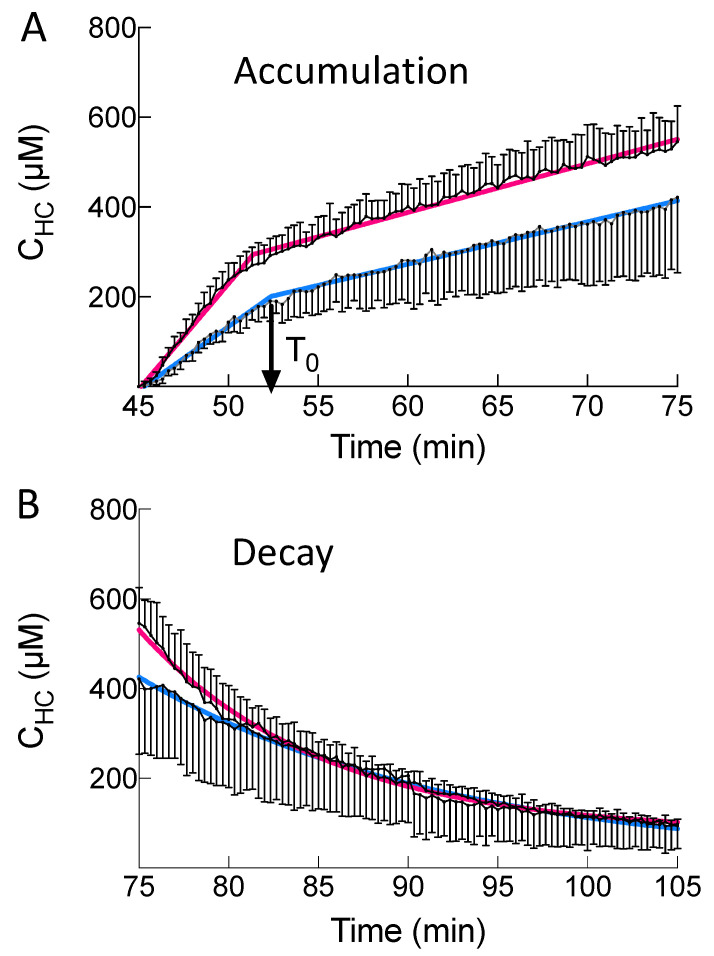
(**A**) Accumulation of BOPTA hepatocyte concentrations from 45 to 75 min. Accumulation was best described by a segmental linear regression (red curve for normal livers and blue curve for MCT livers. T_0_ (black arrows) was the time when BOPTA cellular efflux started to decrease hepatocyte concentrations. T_0_ was similar in both groups. (**B**) BOPTA concentration decay during the rinse period from 75 to 105 min, when livers were perfused only with the Krebs–Henseleit bicarbonate solution. Hepatocyte decay was best described by a one-phase decay (red curve for normal livers and blue curve for MCT livers).

Additional parameters characterise BOPTA accumulation in hepatocytes. The accumulation profile can be described by a segmental linear regression (Figure 5A) [14]. This function defined a first line L_1_ for time below T_0_ and a second line L_2_ for time higher than T_0_, while ensuring that both lines intersect at T_0_. T_0_ is the time when BOPTA efflux from the hepatocytes starts to decrease hepatocyte concentrations. T_0_ occurred 7 ± 1 min (Nl livers) and 8 ± 1 min (MCT livers) after the start of BOPTA perfusion (*p* = 0.41). L_1_ slopes were 47 ± 8 µM/min (Nl livers) and 28 ± 4 µM/min (MCT livers, *p* = 0.02). These slopes characterised BOPTA accumulation into hepatocytes through Oatps. After T_0_, L_2_ slopes were lower than L_1_ slopes (Nl livers: 11 ± 2 µM/min and MCT livers: 9 ± 5 µM/min, *p* = 0.90). In summary, MCT livers had similar T_0_, decreased L_1_ slopes and similar L_2_ slopes than Nl livers. Hepatocyte concentrations (C_HC,100%_) were not significantly different in both groups over time.

At the end of the accumulation period, BOPTA influx clearance (CL_in_) was 2.8 ± 0.4 ml_KHB_/min (Nl livers) and 2.8 ± 0.9 ml_KHB_/min (MCT rats, *p* = 0.90, Table 1). BOPTA hepatic clearance (CL_H_) was 2.4 ± 0.4 ml_KHB_/min (Nl livers) and 1.2 ± 0.4 ml_KHB_/min (MCT livers, *p* = 0.02, Table 1).

### 2.4. BOPTA Elimination from Hepatocytes

To assess BOPTA efflux from hepatocytes, we analysed both the hepatocyte concentration decay and BOPTA recovery in the sinusoids and bile canaliculi. During the rinse period, hepatocyte concentrations were not significantly different over time between both groups (*p* > 0.99, Figure 5B). CL_bile_ and CL_ef_ were defined by the linear regression between v_bile_ or v_ef_ (*Y*-*axis*) and hepatocyte concentrations (C_HC_, *X*-*axis*). CL_bile_ was significantly lower in MCT (0.37 ± 0.17 ml_HC_/min) than in Nl livers (0.93 ± 0.19 ml_HC_/min, *p* = 0.02, Table 1). In contrast, CL_ef_ was significantly higher in MCT (0.75 ± 0.12 ml_HC_/min) than in Nl livers (0.18 ± 0.03 ml_HC_/min, *p* = 0.01). In MCT livers, the high CL_ef_ compensated for the low CL_bile_ and consequently CL_bile+ef_ were similar in both groups (Table 1). This identical CL_bile+ef_ explains the similar decay of BOPTA elimination from hepatocytes in both groups.

The ratios between CL_ef_ and CL_bile+ef_ were 16 ± 3% (Nl livers) and 68 ± 10% (MCT livers, *p* = 0.02). Five min after the start of BOPTA perfusion, bile concentrations (C_bile_) measured in the common bile duct were 859 ± 147 µM (MCT) and 2511 ± 496 µM (Nl) livers (*p* = 0.04). At the end of the accumulation period, C_bile_ remained significantly lower in MCT (9211 ± 1135 µM) than in Nl (16779 ± 2069 µM) livers (*p* = 0.02). The profile of hepatocyte decay was best described by a one-phase decay (Figure 5B). The model measured a rate constant of elimination (k_el,HC_) in MCT (0.10 ± 0.02 min^−1^) that was not significantly different than in Nl livers (0.08 ± 0.02 min^−1^, *p* = 0.25). The apparent hepatocyte volume was not significantly different in MCT (15 ± 1 mL) than in Nl (11 ± 2 mL) livers (*p* = 0.14).

## 3. Discussion

This study evidences a dysfunction of efflux transporters associated with SOS. Other diseases such as liver inflammation, fibrosis, and cirrhosis were previously associated with transporter dysfunction [1,2]. The function of OATPs and MRP2 were altered in human nonalcoholic steatohepatitis, leading to a decreased influx clearance of ^99m^Tc[Mebrofenin] [15]. The biliary clearance of ^99m^Tc[Mebrofenin] was also decreased. The authors anticipated that the pharmacokinetics of drugs transported by OATP1B1/1B3 and MRP2 are likely to have similar abnormalities. SOS induced by pyrrolizidine alkaloids is also associated with biologic markers of cholestasis. High concentrations of total bilirubin were measured following the administration of the pyrrolizidine alkaloid senecionine in rats [16]. Conjugated bilirubin and BOPTA are both transported across hepatocytes by Oatps, Mrp2, and Mrp3/4 transporters [17]. Hyperbilirubinemia is present in patients with SOS [18]. In accordance with our results, hepatic Mrp3 mRNA expression was 13 times higher in senecionine-treated than in normal rats [16]. In contrast, Oatps mRNA expression was significantly decreased in senecionine-treated rats.

As previously published [8], BOPTA bile excretion is altered in livers 18 days after the induction of SOS. Before BOPTA perfusion, bile flow rates in Nl and MCT livers were not significantly different, but BOPTA-induced bile flow increases (choleresis) and BOPTA biliary excretion were importantly reduced in MCT livers during the perfusion period. Similar results were published in mice injected with senecionine [19]. In this study, intravital two-photon imaging showed a delayed biliary excretion rate of fluorescent bile salt analogues. In the present study, we characterised how the alterations of transporter functions in injured hepatocytes explain the decreased biliary excretion of BOPTA associated with SOS.

Besides BOPTA, we perfused the extracellular substrate DTPA to estimate BOPTA extracellular concentrations. BOPTA distribution into the extracellular space was similar in both groups. This result confirmed the absence of fibrosis, inflammation, or hepatocyte necrosis observed in biopsies collected from MCT livers 18 days after SOS induction [8]. Following extracellular distribution, BOPTA enters hepatocytes by Oatps located on the basolateral membrane [11]. The function of Oatps was not modified by MCT according to the similar influx clearances (CL_in_) measured in both groups. However, a case report found a decreased expression of OATP1B3 in a patient with SOS [20]. In the study, the reduced expression was associated with a decreased liver accumulation of another hepatobiliary MRI substrate Gadoxetate. The identical CL_in_ was not associated with a similar early accumulation within hepatocytes (MCT livers had a lower L_1_ slope than Nl livers). One explanation might be that BOPTA sinusoidal distribution is altered in MCT livers, impairing early BOPTA uptake. Moreover, CL_in_ was measured at the end of the perfusion period when hepatocyte concentrations were maximal.

BOPTA biliary clearance (CL_bile_) and BOPTA bile concentrations were much lower in MCT livers, and the choleretic effect of BOPTA was nearly abolished. Mrp2 function is linked to that of aquaporin 8 on cholesterol-enriched domains in the canalicular membrane [21]. However, the mechanism of this link was never investigated. BOPTA efflux clearance back to sinusoids (CL_ef_) was higher in MCT livers and compensated for the low biliary excretion. The total efflux clearance (CL_bile+ef_) was similar in both groups. Thus, Mrp2 function is highly impaired, while that of Mrp3/4 is proportionally increased in MCT livers. Such compensation of CL_ef_ was also observed in livers with steatosis [22]. Similar findings were published with bilirubin [17], morphine [23], and endogenous substrates such as coproporphyrins [24]. Because influx (CL_in_) and efflux (CL_bile+ef_) clearances were similar in Nl and MCT livers, the BOPTA concentration profile in hepatocytes was not significantly different during the perfusion and rinse periods. This cooperation between Mrp2 and Mrp3/4 functions seems useful to maintain normal hepatocyte concentrations. The mechanism that triggers such cooperation was never investigated. In the absence of such cooperation, cholestasis would increase hepatocyte concentrations when Oatp function was maintained.

However, the high BOPTA efflux clearance back to the sinusoids altered BOPTA elimination from MCT livers. Concentrations in the hepatic veins (C_out_) were higher in MCT than in Nl livers because these concentrations include BOPTA concentrations that did not enter the livers plus BOPTA concentrations that had entered the liver but returned to the sinusoids. Liver extraction ratio, sinusoidal removal rates (v), and hepatic clearance (CL_H_) were lower in MCT livers. By extrapolating this finding to the in vivo situation, plasma concentrations would remain higher in MCT than in Nl rats. According to this finding, all substrates including drugs transported by Oatps, Mrp2, and Mrp3/4 would have a decreased plasma concentration decay.

The isolated and perfused rat liver is a convenient model because the experimental conditions are well controlled and simplified. The experimental protocol was designed to measure various parameters during the accumulation and rinse periods. Livers are perfused only through the portal vein with a constant 200 µM concentration of BOPTA. There is no recirculation of solutions, which are discarded after the first pass through the sinusoids. This approach is useful to measure the liver extraction ratio (C_in_ − C_out_)/C_in_. To simplify the protocol, we did not add proteins into the KHB solution, and BOPTA was free to enter the hepatocytes.

The experimental model has limitations. In humans, SOS is associated with more than 20 chemotherapeutic drugs which have no effect on rat livers. However, the rodent syndrome can be induced by pyrrolizidine alkaloids [7,25]. Extrapolation of the results to other species and humans must be cautious because the expression of membrane transporters can differ. We did not measure the expression of transporters but quantified the concomitant functions of three transporters and their resulting effects on hepatocyte concentrations. The experimental model which quantifies all compartmental concentrations, transfer rates, and clearances associated with MCT administration is unique. We improved it over the past decades. The model is adequate for quantifying the changes of BOPTA hepatocyte concentrations associated with various flow rates and drug–drug interactions. Other limitations are linked to the assumptions needed to obtain several pharmacokinetic parameters. BOPTA extracellular concentrations cannot be measured because the substrate enters the hepatocytes within 2 min. To assess these concentrations, we used another MRI substrate, DTPA, which has a similar chemical formula and is distributed only in the extracellular spaces of the liver. Moreover, we assumed that concentrations inside the bile canaliculi were similar to those measured in the common bile duct, although solute export from the cholangiocytes and water transport along the ductules and ducts may modify the primary bile in the canaliculi. This issue was never investigated and has been recently debated by the authors [26]. Another limitation is the ratio between the liver compartments and the liver. The volume ratio of bile canaliculi and liver was previously estimated by Blouin et al. [27] at 0.43% on normal biopsies in rats. In the same article, the volume ratio of hepatocytes and liver was 78%. Because we previously published that fibrosis, inflammation and hepatocyte necrosis disappeared 18 days after MCT administration, we used the same ratios in Nl and MCT livers. CL_bile_ was similar during the accumulation and rinse periods (data not shown), and we assumed that it was also true for CL_ef_ during both periods. Then, we were able to estimate C_ef_ or BOPTA concentrations that returned into the sinusoids by (C_HC_ × CL_ef_)/Q_H_

## 4. Materials and Methods

### 4.1. Induction of the Sinusoidal Obstruction Syndrome (SOS)

Male Sprague–Dawley rats (175–250 g, n = 4) had an intragastric administration of MCT (160 mg/kg, Sigma, Buchs, Switzerland) while 5 additional normal (Nl) rats had an intragastric administration of saline solution [7,28]. MCT livers were isolated and perfused 18 days after gavage. The protocol was carried out in accordance with the Swiss Guidelines for the Care and Use of Laboratory Animals and was approved by the local animal welfare committee and the veterinary office in Geneva, Switzerland (N° 1006.3384.2).

### 4.2. Isolated and Perfused Rat Livers

Before liver isolation, normal (Nl) rats and rats that received MCT were anesthetised with pentobarbital (50 mg kg^−1^, ip). In these two groups, we published preliminary data on the transport of BOPTA across the hepatocyte membranes [8]. In the present study, we returned to the raw data to thoroughly analyse new pharmacokinetic parameters as recently published [29]. Rat livers were isolated, leaving the liver in the carcass. The abdominal cavity was opened, and the portal vein cannulated. The hepatic artery was not perfused. The abdominal vena cava was transected, and an oxygenated Krebs–Henseleit bicarbonate (KHB) solution was pumped into the portal vein, the solution being discarded after liver distribution via vena cava transection. The flow rate was slowly increased over 1 min up to 30 mL/min. In a second step, the chest was opened, and a cannula was inserted through the right atrium to collect solutions flowing from the hepatic veins. Finally, the abdominal inferior vena cava was ligated, allowing solutions perfused by the portal vein to be eliminated by the hepatic veins.

The perfusion system included a reservoir, a pump, a heating circulator, a bubble trap, a filter, and an oxygenator. Solutions of perfusion were equilibrated with a mixture of 95% O_2_ and 5% CO_2_. Livers were continuously perfused with fresh solutions using a nonrecirculating system. The common bile duct was cannulated with a PE_10_ catheter, and bile samples were collected every 5 min to measure bile flow rates (Q_bile_, µL/min per liver) and BOPTA concentrations (C_bile_, µM). BOPTA-increased bile flow rates were measured by the area under the curve of Q_bile_ increases over time (AUCQ_bile_, µL). Samples were collected from hepatic veins every 5 min to measure BOPTA concentrations (C_out_, µM). C_out_ during the BOPTA perfusion period were concentrations that did not enter into the hepatocytes plus concentrations that entered into the hepatocytes and returned to the sinusoids (Figure 1A). During the rinse period, C_out_ were concentrations leaving the hepatocytes because no BOPTA was perfused. To estimate concentrations leaving the hepatocytes during the perfusion period, we used the equation (C_HC_ • CL_ef_)/Q_H_ (see later sections).

A gamma counter was placed over the liver to detect BOPTA concentrations in a single region of interest that must be representative of the entire liver. Concentrations in the region of interest averaged out the concentrations of numerous hepatocytes, knowing that Oatps are mainly expressed in perivenous hepatocytes where BOPTA is likely to enter.

### 4.3. Perfusion of DTPA and BOPTA

Rat livers were perfused with gadopentetate dimeglumine (DTPA; Magnevist^®^; Bayer imaging) and gadobenate dimeglumine (BOPTA, MultiHance^®^; Bracco Imaging). DTPA distributes only within the sinusoids and interstitium, while BOPTA distributes into the extracellular space, hepatocytes, and bile canaliculi. DTPA and BOPTA labelled with ^153^Gd were obtained by adding ^153^GdCl_3_ (1 MBq/mL) to the commercially available (0.5 M) solutions of DTPA and BOPTA. Then, [^153^Gd]DTPA and [^153^Gd]BOPTA were diluted in the KHB solution to obtain a 200 µM concentration. Livers were successively perfused with 200 µM [^153^Gd]DTPA (10 min), KHB solution (35 min), 200 µM [^153^Gd]BOPTA (30 min, accumulation or perfusion period), and KHB solution (30 min, decay or elimination period) (Figure 1B). The protocol lasted 105 min for each group. BOPTA extracellular concentrations cannot be measured because the substrate enters within 2 min into the hepatocytes. To assess these concentrations, we used another MRI substrate DTPA which has a similar chemical formula and distributes only in the extracellular space of the livers. BOPTA and DTPA are widely used in human imaging to characterise BOPTA extracellular and hepatocyte accumulation.

### 4.4. BOPTA Concentrations in Liver Compartments

To quantify BOPTA concentrations in the liver compartments, a gamma counter that collects count rates every 20 s was placed 1 cm above the right liver lobe. The counter measured the radioactivity in a region of interest that was identical in each rat liver. To transform count rates into BOPTA concentrations, the total liver radioactivity was measured by an activimeter at the end of each experiment and was related to the last count rates. Radioactivity was corrected for decay.

The gamma counter delineated a region of interest in the liver lobe from which all count rates originating from the extracellular space, hepatocytes, and bile canaliculi were divided by the liver weight to obtain liver concentrations (C_liver_, µM). Concentrations in the extracellular space were measured during DTPA pre-perfusion (C_EC_, µM). C_EC_ was constant during the 10 min perfusion. We assumed that concentrations inside the bile canaliculi were similar to those measured in the common bile duct (C_bile_, µM), although solute export from the cholangiocytes and water transport along the ductules and ducts might have modified the primary bile in the canaliculi. This issue was recently debated by the authors [26]. The volume ratio of the bile canaliculi and liver was previously estimated by Blouin et al. [27] at 0.43%, and BOPTA concentrations in the bile canaliculi detected by the counter (C_BC_) were 0.0043 × C_bile_. Hepatocyte concentrations (C_HC78%_) detected by the counter were C_liver_ − C_EC_ − 0.0043 C_bile_. Blouin et al. [27] previously determined that the volume ratio of hepatocytes in livers without fibrosis or inflammation was 78%. In situ BOPTA hepatocyte concentrations (C_HC100%_) were calculated by (100/78) × C_HC78%_.

### 4.5. BOPTA Transfer Rates and Clearances between Compartments

BOPTA removal rates from sinusoids during the perfusion period (v, nmol/min) were measured by Q_H_ × (C_in_ − C_out_), where Q_H_ is the constant liver flow rate (30 mL/min), C_in_ (µM) is the constant portal concentration, and C_out_ (µM) is the concentration measured in the hepatic veins. The unbound fraction in the solutions was 1 because no protein was added into the solutions. Hepatic clearance (CL_H_, mL/min) was the ratio of v and C_in_ during the last min of perfusion. The BOPTA extraction ratio (ER) was (C_in_ − C_out_)/C_in_. The BOPTA biliary excretion rate (v_bile_, nmol/min) was C_bile_ × Q_bile_, where C_bile_ (µM) was the concentration in the common bile duct and Q_bile_ was the bile flow rate (µL/min per liver). Clearance from the hepatocytes to the bile canaliculi (CL_bile_, ml_HC_/min) was the slope of linear regression between v_bile_ (*Y*-axis) and the hepatocyte concentrations (C_HC_, *X*-axis), which was measured during the entire protocol. This clearance was expressed in ml of hepatocytes (ml_HC_) per min.

During the rinse period, BOPTA concentrations leaving the hepatocytes (C_ef_, µM) into the sinusoids were measured by C_out_ because no BOPTA was perfused in the portal vein. Basolateral efflux from the hepatocytes into the sinusoids (v_ef_, nmol/min) was C_ef_ × Q_H_, and basolateral clearance (CL_ef_, ml_HC_/min) was the slope of linear regression between v_ef_ (*Y*-axis) and C_HC_ (*X*-axis). With the assumption that CL_ef_ was similar during both perfusion and rinse periods, we estimated C_ef_ during the accumulation period by (C_HC_ × CL_ef_)/Q_H_. The hepatocyte influx rate v_in_ (nmol/min) was [C_in_ − (C_out_ − C_ef_ )] × Q_H_ or v + v_ef_. In other words, BOPTA entry into the hepatocytes includes BOPTA eliminated into the bile canaliculi and back into the sinusoids. Hepatocyte influx clearance CL_in_ was v_in_/C_in_. CL_in_ was measured during the last min of perfusion and is expressed in ml of Krebs–Henseleit bicarbonate solution per min (ml_KBH_/min)

### 4.6. Accumulation Profile of Hepatocyte Concentrations

During the perfusion period, BOPTA hepatocyte accumulation was best described by a segmental linear regression obtained from GraphPad Prism version 8, GraphPad Software, La Jolla, CA, USA [14]. This function defines a first line L_1_ for time below T_0_ and a second line L_2_ for time higher than T_0_. T_0_ is the time when BOPTA efflux from the hepatocytes decreases BOPTA accumulation into the hepatocyte. No constraint was applied to fit the data.

### 4.7. Decay Profile of Hepatocyte Concentrations

During the decay period, the data were best described by a one-phase decay (GraphPad Prism version 8, GraphPad Software, La Jolla, CA, USA) [30]. No constraint was applied to fit the data. The model was defined by a rate constant of elimination (k_el,HC_, min^−1^). Knowing CL_bile+ef_ and k_el,HC_, we calculated the apparent hepatocyte volumes (V_HC_, mL) as CL_bile+ef_/k_el,HC_.

### 4.8. Statistics

Data are means ± SD. Parameters obtained in Nl and MCT livers were compared with a Mann–Whitney test (GraphPad Prism version 8, GraphPad Software, La Jolla, CA, USA). To compare over time the effect of treatment (Nl vs. MCT) on additional parameters, we used a two-way ANOVA with Sidak multiple comparison tests. *p* < 0.05 was considered statistically significant.

## 5. Conclusions

The study shows that SOS is associated with a long-lasting altered function of the efflux transporters Mrp2 and Mrp3 in hepatocytes. BOPTA hepatocyte influx clearance was similar in both groups, while biliary clearance and bile concentrations were much lower in MCT than in Nl livers. BOPTA efflux clearance back to sinusoids compensated for the low biliary excretion and consequently, hepatocyte concentrations remained similar in both groups. This SOS-associated changes of transporter functions might impact the pharmacokinetics of numerous drugs that use similar transporters to cross the hepatocytes.

## Figures and Tables

**Table 1:** BOPTA values at the end of the accumulation period at time 75 min.

Livers	Nl	MCT	*p*
*BOPTA concentrations*			
C_in_ (µM)	200	200	
C_out_ (µM)	184 ± 2	192 ± 2	0.02
C_EC_ (µM)	56 ± 11	68 ± 29	0.56
C_BC_ (µM)	72 ± 9	40 ± 5	0.02
C_HC78%_ (µM)	425 ± 63	329 ± 131	0.41
C_liver_ (µM)	551 ± 65	436 ± 155	0.41
C_bile_ (µM)	16,779 ± 2069	9211 ± 1135	0.02
*BOPTA transfer rates*			
v (nmol/min)	472 ± 70	246 ± 70	0.02
v_in_ (nmol/min)	567 ± 74	554 ± 178	0.90
v_bile_ (nmol/min)	404 ± 64	100 ± 32	0.02
v_ef_ (nmol/min)	96 ± 12	308 ± 114	0.02
v_bile+ef_ (nmol/min)	500 ± 67	409 ± 121	0.41
*Clearances*			
CL_H_ (ml_KHB_/min)	2.4 ± 0.4	1.2 ± 0.4	0.02
CL_in_ (ml_KHB_/min)	2.8 ± 0.4	2.8 ± 0.9	0.90
CL_bile_ (ml_HC_/min)	0.93 ± 0.19	0.37 ± 0.17	0.02
CL_ef_ (ml_HC_/min)	0.18 ± 0.03	0.75 ± 0.12	0.02
CL_bile+ef_ (ml_HC_/min)	1.11 ± 0.21	1.12 ± 0.23	0.99
CL_ef_/CL_bile+ef_ (%)	16 ± 3	68 ± 10	0.02

Concentrations in portal vein (C_in_), hepatic veins (C_out_), extracellular space (C_EC_), and bile (C_bile_). Concentrations measured by the counter: liver (C_liver_), hepatocytes (C_HC78%_), and bile canaliculi (C_BC_). Substrate removal rate from sinusoids (v), hepatocyte uptake rate (v_in_), bile excretion rate (v_bile_), and efflux rate back into sinusoids (v_ef_). CL_H_ (hepatic clearance), CL_in_ (hepatocyte uptake clearance), CL_ef_ (basolateral clearance) and CL_bile_ (biliary clearance) are expressed in ml_KHB_/min or ml_HC_/min. Volumes in ml are Krebs–Henseleit bicarbonate (KHB) solution or hepatocytes (HC).

## Data Availability

The data presented in this study are available on request from the corresponding author.
